# Exopolysaccharides Producing Lactic Acid Bacteria in Wine and Other Fermented Beverages: For Better or for Worse?

**DOI:** 10.3390/foods10092204

**Published:** 2021-09-17

**Authors:** Maria Dimopoulou, Marguerite Dols-Lafargue

**Affiliations:** 1 Department of Wine, Vine and Beverage Sciences, School of Food Science, University of West Attica, Ag. Spyridonos str, Egaleo, 12243 Athens, Greece; mdimopoulou@uniwa.gr; 2 Unité de Recherche Œnologie EA 4577, University of Bordeaux, ISVV, USC 1366 INRA, Bordeaux INP, F-33140 Villenave d’Ornon, France

**Keywords:** lactic acid bacteria, wine, exopolysaccharides, fermented beverages

## Abstract

Lactic acid bacteria (LAB) from fermented beverages such as wine, cider and beer produce a wide range of exopolysaccharides (EPS) through multiple biosynthetic pathways. These extracellular polysaccharides constitute key elements for bacterial species adaptation to such anthropic processes. In the food industry, LAB polysaccharides have been widely studied for their rheological, functional and nutritional properties; however, these have been poorly studied in wine, beer and cider until recently. In this review, we have gathered the information available on these specific polysaccharide structure and, biosynthetic pathways, as well as the physiology of their production. The genes associated with EPS synthesis are also presented and compared. Finally, the possible role of EPS for bacterial survival and spread, as well as the risks or possible benefits for the winemaker and the wine lover, are discussed.

## 1. Introduction

Lactic acid bacteria (LAB) form a large group of bacteria, and are widely used throughout the world for the biotransformation of animal and plant resources. From very early on, they were valued to preserve and improve the organoleptic or nutritional properties of many foods. This is also the case in fermented beverages, where many distinct genera and species have been described [1]. However, not all species are found in all beverages. Species distribution seems rather specific to a particular type of fermented drink. Furthermore, LAB are sometimes hardly detectable in fruits and become dominant at certain steps in the production process. In fact, in wine and cider, LAB develops after the yeasts first drive alcoholic fermentation (AF). At the end of AF, upon lysis of the yeasts, the medium contains high levels of ethanol and very few residual sugars; it is acidic. Only certain species have been found to resist this process, including LAB of the genera *Lactobacillus*, *Pediococcus*, *Oenococcus* and *Leuconostoc* [2]. These bacteria multiply by consuming the elements left by the yeasts (sugars, acids). Depending on the metabolic pathways taken to break down the sugars, they produce either only lactic acid (homofermentative bacteria) or a mixture of lactic acid and acetate or ethanol (heterofermentative bacteria) [2]. However, the main visible transformation carried out by lactic acid bacteria in wine and cider is the conversion of the L-malic acid present into L-lactic acid and CO_2_. Although this is not fermentation *sensu stricto*, this reaction is called malolactic fermentation (MLF). MLF is important for three reasons [3]: (i) the transformation of malic acid (di-acid) into lactic acid (monoacid) softens the beverage, reducing its total acidity; (ii) the growth of bacteria is accompanied by the consumption of the residual substrates of the medium, which limits the risk of development of the spoilage flora during storage and aging and (iii) the release of aromas (from grapes, apples, yeasts or wood precursors) and the production of secondary metabolites by bacteria contribute to the final sensorial properties of the wine [4,5,6] In the same way, LAB also modulate the ciders final quality [7,8].

The success of MLF relies on the ability of indigenous LAB to survive and develop under particularly hostile physicochemical conditions. Only the most resistant LAB are selected. Though MLF can also be carried out by genera other than *Oenococcus* [4,9] most often, *Oenococcus oeni* becomes the dominant species and the main driver of MLF in temperate-zone wines or ciders, although it is hardly detected on the fruits [4,8,10]. The bacterial strains found in ciders and wines are different, suggesting that a form of domestication has occurred within the species. Other species of the genus *Oenococcus* are found in fermented products made from sugar cane (*O. alcolitolerans* [11]) or during the preparation of schochu (*O. kitaharae* [12]). *O. sicerae* is found specifically in ciders [13].

Certain particularly efficient LAB strains have been selected and are marketed as malolactic starters [9]. Conversely, certain strains of LAB (including *O. oeni*) can cause deterioration before, during or after MLF, depending on their genome content and the associated metabolic abilities [14]. Furthermore, the context (type of beverage or even type of wine considered) may also modulate the microbial metabolic activities and thus the risk associated with the presence of a specific strain. The machinery necessary for the biosynthesis of exopolysaccharides (EPSs) is one of the metabolic tools which differentiates the LAB strains. EPS can (i) contribute to bacterial survival in the specific context of the production of fermented drinks, (ii) improve the sensory properties of wines and other fermented drinks or (iii) on the contrary, lead to beverage spoilage [4,15,16].

The aim of this review is to synopsize the current knowledge on the EPSs produced by the LAB of fermented drinks, namely, the nature and location of the EPSs, the genes involved and the active biosynthetic pathways, as well as the consequences of EPS production on the survival of the bacteria and on the quality of the beverages produced. Finally, research perspectives will be examined.

## 2. EPSs Produced and Biosynthetic Pathways

EPSs are extracellular glucidic polymers of variable size (a few tens of monosaccharides to several tens of thousands). They may consist of a single type of monomer (homopolysaccharides) or of several different monomers (heteropolysaccharides), they may be neutral or charged and may or may not contain non-carbohydrate substituents. Finally, they can be linear or branched. Among fermented drinks, the LAB EPSs *O. oeni* EPSs are the ones that have been the most studied—data on this species will therefore be presented first, and where possible, knowledge will be extended to other species (Table 1).

### 2.1. EPSs Produced by Wine and Cider LAB in Brief

The β-1,3 β-1,2 glucan was the first EPS produced by wine or cider LAB to be identified [21]. It comprises a backbone made of β-1,3 linked glucoses and branches made of a single β-1,2 linked glucose attached to each of the two or three residues of the main chain. This polymer will be referred to as “β-glucan” throughout this review. Its accumulation in beverages induces an increase in viscosity and even a ropy character: the wine, beer or cider can harbor an oily texture, even when β-glucan concentrations are as low as 12 mg/L [38]. Many bacterial species are able to produce this specific glucan: *O. oeni*, *Lactobacillus suebicus*, *Lactobacillus diolivorans*, *Pediococcus parvulus*, *Pediococcus damnosus*, *Lactobacillus collinoides*, *Lactobacillus brevis*, *Lactobacillus rossiae*, *Lactobacillus parabuchneri* and *Levilactobacillus brevis* [17,18,20,23,26,27,28,30,37,39,40]. Additionally, heteropolysaccharides, dextrans and fructans are also produced by *O. oeni* and some other species [38].

Many wine or cider bacteria have been shown to produce several types of polymers simultaneously. For example, certain strains of *O. oeni* isolated from wine or cider produce both dextran (α-1,6-α-1,3 glucan), levan (β-2,6 fructan), β-glucan and heteropolysaccharides composed of galactose, glucose and rhamnose [35]. *Pediococcus ethanolidurans* isolated from Basque cider also produces both ropy β-glucan and a heteropolysaccharide composed of glucose, galactose, glucosamine and glycerol-3-phosphate [24].

However, depending on the conditions, one of the polymers formed is much more abundant than the others and explains the dominant phenotype of the strain: capsulated, mucous, ropy or sticky [35,41]. The production could be modulated by the presence of numerous phenolic compounds in these beverages [42]. Furthermore, in wine or grape juice, the concentrations of the EPS produced are often hardly noticeable due to the presence of numerous other polysaccharides liberated from the grapes and yeasts [43,44].

### 2.2. EPS Localization

EPS can be classified into three major groups, according to their exact external location [45]:

- WPS or wall polysaccharides, attached to the cell, covalently or not, but without forming a capsule.
- CPS (or capsular polysaccharides), most of the time linked to peptidoglycan, forming either a thick and cohesive (capsule) or a thin and cohesive (film) outer layer.
- Exocellular polysaccharides (or true EPSs), released into the environment surrounding the cell during planktonic growth. This kind of true EPS can also form a slime or a polymeric matrix during growth on solid media or biofilm formation.

The distinction between these polysaccharides is sometimes controversial. The capsules are observable in negative staining in classical microscopy, but some polymers can form a dense layer that is visible in electron microscopy but not thick enough to be visible in negative staining [46,47]. In addition, CPS can be released depending on the growing conditions or due to unstable cell binding and can be mistaken for EPSs. Conversely, certain EPSs can be found linked to the cell and to the peptidoglycan, even non-covalently [46].

For instance, most *O. oeni* strains studied are encapsulated by a heteropolysaccharidic layer and this kind of “capsule” seems to be present regardless of the culture conditions. However, some of these CPSs are released into the surrounding medium at the end of the stationary phase [35] or during growth on solid media [27]. On the other hand, the capsular β-glucan of *P. parvulus* 2.6, a ropy strain isolated from cider, can be released from the cell “capsule” into the medium by means of simple cell washes [48].

### 2.3. Biosynthetic Pathways

The EPS biosynthetic pathways involving sugar nucleotides have been extensively studied in milk LAB (*Streptococcus thermophilus*, *Lactobacillus rhamnosus* and *Lactococcus lactis*) and in pathogenic streptococci (*Streptococcus pneumonia* and *Streptococcus agalactiae*) [49,50,51,52,53,54]. Dextran and levan synthesis have been mainly studied in *Lc. mesenteroides* [55].

**Figure 1:**
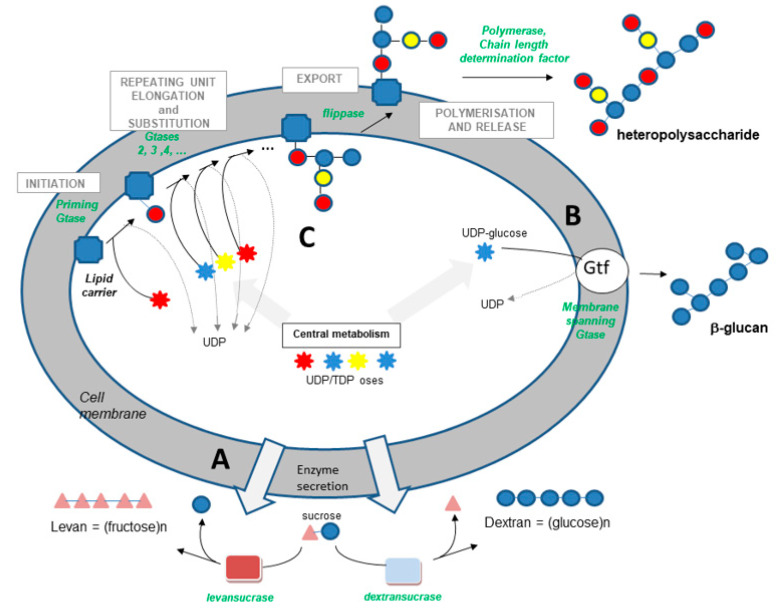
Overview of the EPS biosynthetic pathways active in *O. oeni* and other LAB from fermented beverages. From top to bottom and right to left: (**A**) Dextran (α-glucan) and levan (β-fructan) synthesis from sucrose by external glycansucrases such as dextransucrase or levansucrase. (**B**) β-glucan synthesis involving UDP-glucose and the membrane spanning glycosyltransferase Gtf. (**C**) Heteropolysaccharide synthesis: the repeating unit is built via the involvement of priming glycosyltransferase and several other glycosyltransferases, a lipid carrier and activated substrates (UDP-oses). Then, the complete repeating unit is externalized by a flippase and is polymerized outside the cell before the release of the polymer. Gtase, glycosyltransferase.

Similar pathways were found to be active in LAB of wine and fermented beverages. Actually, in these specific bacteria, three types of cellular machinery dedicated to the biosynthesis of EPSs have been described to date (Figure 1): (i) extracellular transglycosidases called glycansucrases, which use sucrose as a substrate and catalyze the synthesis of homopolysaccharides (α-glucans, β-fructans); (ii) isolated synthases that use nucleotide sugars as substrates and which alone catalyze the polymerization and export of homopolysaccharides; and (iii) complex systems involving nucleotide sugars as substrates and many enzymes that achieve the synthesis and export of complex heteropolysaccharides together [38].

(i) Transglycosidases, which specifically use sucrose as a substrate (or glycansucrases), are classified into the CAZy GH-13, 68 and 70 families (www.cazy.org (accessed on 14 September 2021)) [56]. They catalyze the synthesis of homopolysaccharides made up of glucose or fructose, according to the following simplified reactions:

n sucrose → (fructose)n + n glucose (fructansucrase)

n sucrose → (glucose)n + n fructose (glucansucrase)

These enzymes are exocellular (Figure 1). They catalyze the synthesis of polysaccharides via a succession of donor-acceptor type reactions (a processive mechanism [57]). They are produced by certain strains of different species found in fermented beverages (*O. oeni*, *O. kitaharae*, *Lc. mesenteroides*, *Lc. pseudomesenteroides*) [32,33,35,41,58]. The structure of the polymer produced varies depending on the producing enzyme, both in terms of the type of osidic bonds formed (α-1,6 and α-1,3 in varying proportions for glucans and mainly β-2,6 for fructans), and in terms of degree of polymerization. Interestingly, the dextran produced by the enzyme DsrOK from *O. kitaharae* is one of the largest ever studied [58]. The enzyme also displays a very high catalytic efficacy, whereas the enzyme DsrO of *O. oeni* is poorly efficient and quite unstable [34,58].

(ii) The synthase pathway involves a single membrane-spanning enzyme, which alone carries out the initiation of synthesis, the elongation of the polymer (processive enzyme) and its export through the membrane (Figure 1). The β-glucan causing wine or cider ropiness is produced by an enzyme of this type, called Gtf. The role of Gtf in the synthesis of *P. parvulus* and *O. oeni* β-glucan was demonstrated in 2006 and 2008 [17,18]. Gtf is 32% identical to Tts, a synthase found in *S. pneumoniae* type 37, which produces a β-glucan with structure close to that produced by wine, beer and cider strains. This pneumococcal glucan is immunogenic in humans, as in mice, and was shown to be responsible for pneumococcal strain virulence [59].
(iii) The third pathway is the most complex (Figure 1). This pathway is sometimes called the Wzy-dependent pathway, based on the name given to the polymerase in *E. coli* [60]. This pathway has been perfectly characterized in Gram-negative bacteria and partially in Gram-positive bacteria [49,50,51,52,60]. The first step is the synthesis of a repeating oligosaccharidic unit through the transfer of monomers to a lipid transporter on the inner face of the cell membrane (Figure 1). This synthesis, carried out by a series of non-processive glycosyltransferases, is followed by the export of the repeating unit by a flippase (Wzx) and by the assembly of the exported repeating units by a polymerase attached to the external face of the cell membrane (Wzy). Regulating enzymes and factors modulate the chain length and the polymer release. The glycosyltransferase which initiates the synthesis of the repeat unit by transferring the first monomer to the lipid transporter is called the “priming glycosyltransferase”. Several priming glycosyltransferases are found in *O. oeni* and complement each other [61], ensuring EPS formation even in cases in which mutations inactivate one of the enzymes.

The distinction between biosynthetic pathways using a transglycosidase and sucrose as a precursor or glycosyltransferase(s) and sugar nucleotides is very important from a physiological point of view—the biosynthetic pathways involving sugar nucleotides are very “expensive” for the bacteria from an energetic point of view. They compete for nucleotide sugars with the cell-wall synthetic pathways, which limits the production levels of liberated polymers to a few hundred mg/L, whereas the production levels can rise to a few tens of grams per liter, in the case of glucansucrase using sucrose [35,41,44,62]. The *O. oeni* strains equipped with active glucansucrase or fructansucrases release from 0.2 to 8 g/L of polymers depending on the strains and growing conditions. Sucrose has to be added to the growth media [34,35]. In the presence of high sucrose concentrations, *Lc. mesenteroides* isolated from Spanish wines can produce 500 mg/L of dextran [33].

The Wzy-dependent pathway leads to the release of at most 250 mg/L of heteropolysaccharide in *O. oeni* and about 50 mg/L in *P. ethanolidurans* [24,35,44]. Furthermore, depending on the bacterial strain and the growth conditions, the concentration of β-glucan can vary between 10 and 250 mg/L [18,19,44].

### 2.4. Genes Associated with EPS Synthesis

Glycansucrases and autonomous synthase, which are enzymes capable of catalyzing the synthesis of polymers on their own, are generally encoded by isolated genes, whereas Wzy-dependant pathways are generally encoded by genes in large clusters or operons. The *eps* genes can be chromosomal or plasmidic. In wine and cider LAB, some of these genes seem extremely mobile, in particular the *gtf* gene, associated with the synthesis of the β-glucan causing wine or cider ropiness. In fact, it is carried by at least four different plasmids within the genus *Pediococcus* [17,23,24,63], and it is chromosomic (but inserted in a phage remnant or even in a prophage) in *O. oeni* [18,35]. Despite the high number of genetic locations, the gene is more than 95% conserved between the bacterial species capable of producing the ropy β-glucan: *Lb. suebicus*, *Lb. diolivorans*, *P. parvulus*, *P. damnosus*, *Lb. collinoides* and *O. oeni* [17,18,23,28,30,37,39,40]. This suggests either a recent transfer between the bacteria or a high-level conserved amino acid sequence requirement for maintaining the activity.

Dextransucrase genes are generally chromosomic and highly conserved in *O. oeni*, even though the main habitat of the species, wine, does not contain any sucrose. Truncated forms are sometimes found but most of the time the *dsrO* gene is not truncated [35]. This nearly ubiquitous presence of a very large dextransucrase gene (>3000 bp) suggests that dextransucrase activity is important for the survival of the species, but this may be in a context other than wine. Other *Oenococcus* species also display dextransucrase genes, except in *O. kitaharae* these have not yet been characterized [58].

**Figure 2:**
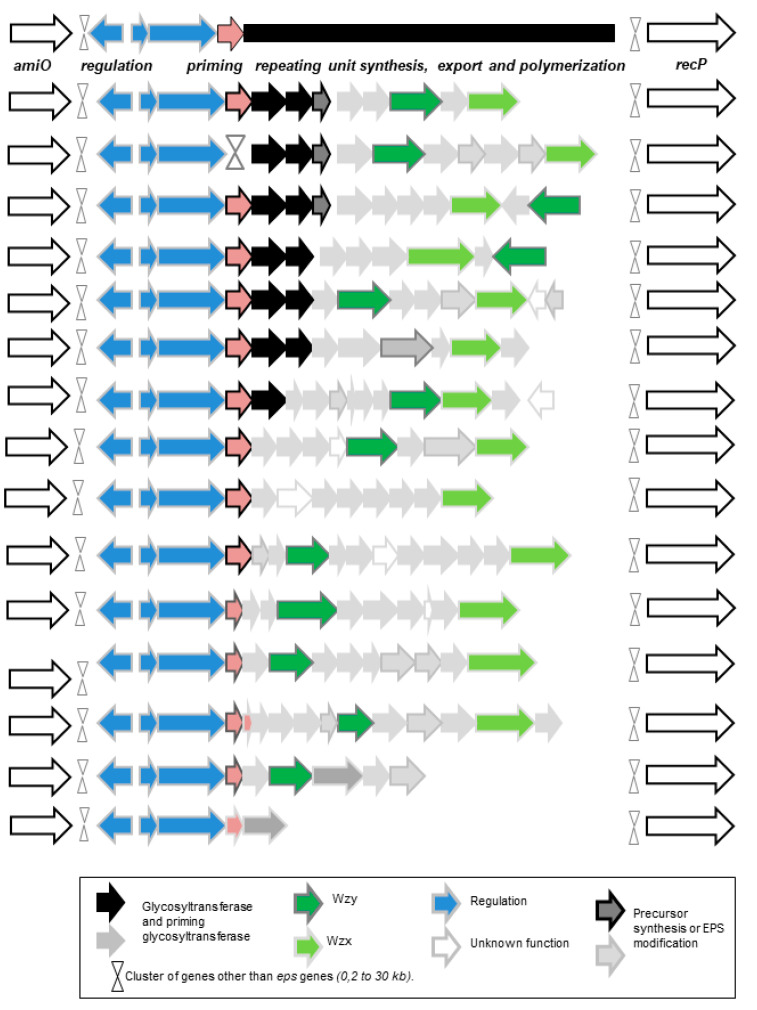
Consensus organization of *eps* gene cluster 2 in *O. oeni* (adapted from Dimopoulou et al. [35]).

In LAB, the genes encoding the enzymes involved in the Wzy-dependent pathway are organized in clusters or operons, in which regulatory genes or genes for the synthesis of precursors are often found [35,64,65]. These clusters still exhibit a high density of coding zones and are chromosomic in the fermented-drink LAB strains studied to date. In *O. oeni* the *eps* gene cluster organization is often unidirectional (Figure 2). The 5′ region is the most conserved one and it displays regulatory genes, the priming glycosyltransferase gene, then the genes encoding the other glycosyltransferases, together with genes involved in precursor biosynthesis, followed by *wzy* and *wzx*. Dimopoulou et al. found 14 distinct complete *eps* clusters out of 50 *O. oeni* genomes studied [35]. Recombination events are certainly at the origin of the final diversity observed. These recombination events may lean on the conserved 5′ region of the *eps* gene cluster and on the 3′ end, on the gene *recP* (Figure 2). The size of the chromosome region concerned in the recombination process can be as high as 50 kb. Such a diversity of *eps* gene clusters is often described as a component of adaptation to host defense mechanisms in pathogenic bacteria [66,67,68]. Actually, more than 88 wzy-dependent gene clusters, causing as many serotypes, have been described in *S. pneumoniae* [65].

To sum up, in the O. *oeni* chromosome, regardless of the strain considered, there is at least one gene dedicated to EPS synthesis which encodes a functional pathway, and very often several genes encode several active pathways [35]. However, the selective advantage of such a high diversity of *eps* genes and clusters for *O. oeni*, a non-pathogenic bacterial species, remains unclear. This point will be further discussed in the following paragraphs.

This *eps* gene cluster is present in more than 85% of the *O. oeni* strains studied and it is associated with capsular polysaccharide synthesis. Only the last one presented in the bottom of this figure encodes an inactive pathway (no CPS produced; the gene transcription was not examined). The cluster is highly variable, especially in its 3′ end, whereas the 5′ end sequence is more conserved. The genes *wzx* and *wzy* are the most divergent ones and appear to be specific to each complete cluster. Several recombination events upstream and downstream of the *eps* locus may have led to the diversity observed. These events may lean on *recP* or the cluster of genes inserted between *recP* and the 3′ end of the *eps* gene cluster, or even on the *eps* gene cluster itself. In parallel, recombination events may have occurred upstream of *eps* gene cluster 2, between the genes of *amiO* and *wze* or *wzd*.

## 3. What Could Be the Consequences for the Winemaker or the Wine Lover?

**Figure 3:**
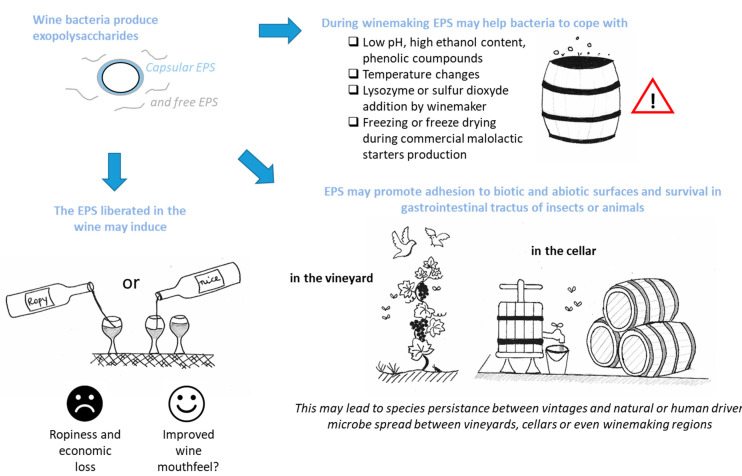
Possible incidence of bacterial polysaccharides in the specific context of winemaking.

The exact role of polysaccharides in bacterial physiology is not perfectly understood. Nevertheless, the fact that the majority of the wine microorganisms have the ability to produce several EPS structures independently of the genus, species or strain could indicate the importance of these polymers for the survival of the individuals or for maintaining the species. The production of fermented beverages from fruits is a seasonal activity. Microorganisms must therefore survive not only during the beverage production process but also, once the beverages are produced, in cellars or factories and/or in orchards and vineyards for almost a year. LAB may therefore be able to survive in the gastrointestinal tract of insects and birds that gravitate to production areas and/or on production equipment or on trees, plants and soils, which would promote (1) their survival between seasons and (2) their natural spread or their human-driven spread (wine trade, exchange of winemaking materials, Figure 3).

### 3.1. EPSs for Bacteria Survival in Harsh Winemaking Conditions

The presence of an extracellular polymeric matrix could be implicated in the protection of the cell in various situations including wine or cider elaboration [69,70]. Indeed, many bacteria enhance EPS synthesis as a response to carbon dioxide release by yeasts during AF [71,72] or in the case of various stresses such as low pH or high ethanol or phenolic compound concentrations [23,34]. For example, β-glucan seems to increase the resistance of the producing bacteria to acidic pH [18,27,48]. In France, the prevalence of ropy *O. oeni* strains is particularly high in regions producing naturally more acidic wines (Bourgogne or Champagne) [18,73,74]. The increase in viscosity induced by β-glucan may slow down the diffusion of abiotic compounds, such as phenolic compounds, sulfites, and ethanol, and protect the bacteria. By constituting a protective layer, β-glucan is also involved in the resistance of LAB to lysozyme treatment [75]. LAB CPSs and dextrans can also offer protection during the freeze-drying step in malolactic starter production [34,76]. On the other hand, the phage resistance associated with EPSs is controversial and EPSs could, depending on the case, serve as a target for attachment and recognition or, on the contrary, as a masking element for these targets [77,78].

### 3.2. EPSs, Biotic Interactions and Species Survival

Beta-glucan was shown to promote the adhesion of bacteria to intestinal cells and to play a role in cross feeding in the intestinal tract, and this may promote microbial survival in the intestinal tracts of various animal and insects, and hence in bacterial dissemination and the colonization of new ecological niches [48,79,80,81]. In the same way, the high diversity of capsular heteropolysaccharides produced by *O. oeni* may be involved in bacterial survival in a biotic context, through interactions or adhesion to epithelial receptors in the gastrointestinal tracts of insects or animals. The capsule may also constitute a protection against host defense mechanisms and promote host colonization, as shown for many pathogenic Gram-positive and Gram-negative bacteria [66,82,83]. Nevertheless, further work will be necessary to identify the hosts in which wine LAB are accommodated between two vintages, if any.

*O. oeni* glucansucrase genes are highly conserved, despite the fact that the encoded enzymes use a substrate, sucrose, that is rarely found in wine but is present in grapes. The production of dextran on grapes may assist the attachment of the bacteria to fruits or to vine woods. It may also promote their survival in the soil, as suggested a long time ago for *Lc. mesenteroides* [84]. Furthermore, EPS-producing LAB adheres better to *S. cerevisiae* cells, which efficiently consume lactic acid and consequently decrease the acidity of the medium, subsequently promoting bacterial growth [85,86].

### 3.3. EPSs and Bacterial Colonization of the Production Cellar

EPSs may help the wine microorganism to invade during all the winemaking steps through promoting adhesion onto abiotic surfaces, i.e., stainless steel, plastic and glass surfaces, as well as the wood of oak barrels. Undoubtedly, the presence of EPSs modulates the physicochemical characteristics of the LAB cell surface [87]. In some cases, EPSs were shown to be implicated in the adhesion process [88,89]. Adhered cells can, in the second phase, evolve into biofilms. Biofilms are complex structures composed mainly of an extracellular matrix, genetic material and microorganisms. Depending on the case, abundant EPSs can increase or decrease the adhesion capacity and biofilm formation [90].

The same could be assumed for the EPSs produced by wine LAB, i.e., dextran, β-glucan and heteropolysaccharides. EPSs may be important for *O. oeni* biofilm settlement as the genes implicated in heteropolysaccharide and homopolysaccharide production are overexpressed under stressed biofilm conditions [36]. More recently, it has been demonstrated through FT-IR spectra analysis that the metabolic fingerprint of the attached cells was significantly different from that of the planktonic ones, and the spectral zone associated with polysaccharides was discriminative. This suggests the importance of EPSs in biofilm formation on stainless steel surfaces [91]. The β-glucan produced by *O. oeni* or recombinant *L. lactis* expressing *gtf* may be involved in the last steps of adhesion and biofilm consolidation, rather than primary adhesion [18]. Regarding *O. oeni* dextran, the research carried out so far has not made it possible to determine whether it promotes or whether it prevents attachment to abiotic surfaces. This may depend on the strain and the context in which the cells develop. Nácher-Vázquez et al. [92] compared the biofilm formation and aggregation ability of two different bacterial species, *Lc. mesenteroides* and *Lb. sakei*, both of which are great producers of dextrans. Interestingly, only *Lb. sakei* formed a biofilm on the tested abiotic surfaces, whereas dextran exerted an antiadhesive effect in the case of the strain of *Lc. mesenteroides*. Thus, the authors proposed that the role of dextran was involved in the aggregation process and biofilm consolidation, rather than in the primary adhesion process, which is rather linked to the surface proteins of the bacteria. Dextran, which is a neutral and weakly adhesive polymer, may aid in the attachment process by masking repulsion molecules. By contrast, it could prevent adhesion by masking motifs essential for primary adhesion [93,94].

The most important point is that biofilms are a strategy used to overcome stressful conditions [95]. *O. oeni* cells attached to stainless steel and oak wood surfaces can perform malolactic fermentation and display higher tolerance to wine stress than planktonic ones [36]. In the same way, the biofilm cells of *Lb. plantarum* expressed higher resistance to acetic acid and low pH values than the planktonic cells [69].

### 3.4. Beverage Spoilage and Possible Treatments

Some of the EPSs from wine bacteria are implicated in a specific form of wine spoilage, the disease known as “ropiness”. The polymer produced increases the wine’s viscosity and sometimes induces an oily sensation [96]. This ropy character may be perceived with the naked eye and can occur in stainless-steel tanks, in barrels and even in the bottle. If the alteration remains at bottle opening, the consumer who discovers the defect sometimes definitively turns away from the wine from the area concerned and this may provoke great losses for the wine industry (Figure 3).

For the moment, the only molecule clearly implicated in ropiness is the β-glucan described in the previous paragraph, but other molecules could be implicated in other bacteria [23]. Interestingly, wine LAB displaying the ropy phenotype in laboratory media may be isolated from non-ropy wines [44]. This observation indicates that the effect of EPSs on wine depends on many factors and the presence of a “ropy” strain is not sufficient to provoke wine spoilage. In the first place, spoilage is not correlated with EPS quantity but mainly with EPS structure, as just 12 mg/L of β-glucan may provoke ropiness, whereas some g/L of dextran do not induce such a viscosity change. In addition, the role of the wine or cider matrix is essential as interactions of the polymer with the matrix may enhance the viscosity. Interestingly the *Lactobacillus* and *Pediococcus* strains producing β-glucan are also implicated in beer and cider spoilage [20,26,39,97]. *P. parvulus* is the species that is most often involved in the ropiness of wine and ciders, as it develops after fermentation processes in the late stages of winemaking, even after bottling. *O. oeni* ropy strains are quite frequent but, as they develop in the early stages of winemaking, when wine supervision is easier, they are less dangerous for the final quality of the wine (Table 1). Indeed, the vigorous stirring of the wine is enough to make the ropy character disappear and a close supervision of the microbial flora during the following stages of winemaking may guarantee that the viscosity remains low. The well-developed qPCR protocols for the detection of ropy strains, through the detection of *gtf* genes, constitute an interesting tool for wine supervision, early reaction and curative or preventive treatments [17,19,98]. However, LAB spoilage species could be resistant to SO_2_ treatment, as well as to lysozyme [18,75]. Nevertheless, the ropy strains could be remedied via the simultaneous enzymatic action of β-glucanase and lysozyme, as recommended by Coulon et al. [75]. A combined treatment of low concentrations of enterocin AS-48, along with high-intensity pulsed-electric field (HIPEF) treatment, was also shown to inactivate exopolysaccharide-producing LAB strains in apple juice, but these methods need to be investigated more and are not currently allowed [99,100,101].

### 3.5. Bacterial EPSs and Wine Sensorial Properties

For years, bacterial polysaccharides were considered to have no impact on wine, except for β-glucan accumulation and ropiness (see previous paragraph). However, bacterial polysaccharides accumulate in wine during MLF and later even when no viscosity increase is noticeable [35,43]. These biopolymers could directly or indirectly have an impact on the wine’s sensory profile, as previously shown for yeast mannoproteins (MPs) [102,103,104]. In fact, MP enrichment of wines have been proven to modify wine astringency and sucrosity [105,106]. MPs have also been mentioned to play an important role in tartrate salt crystallization, although the MP concentration needs to be adjusted depending on the wine considered [107,108,109]. Additionally, recent studies have shown that MPs can interact with all anthocyanin families and modulate the bioaccessibility of polyphenolic compounds during digestion in the gastrointestinal tract [110]. In addition, pectin, a heteropolysaccharide mainly composed of partially methylated galacturonic acid, can inhibit proteins and tannin precipitation [111]. These interactions also depend on the hydrophobicity and molecular weight of the phenolic compounds [112]. In the same way, the EPSs liberated by LAB could interfere with tannins and salivary proteins and thus reduce the sense of astringency. This could be the case with *O. oeni* dextrans. Indeed, small commercial dextrans (>5000 da) were shown to increase the mouthfeel sensation and to decrease wine astringency (Dimopoulou et al., unpublished).

Even if polysaccharides are not volatile molecules, they may indirectly contribute to aroma perception. Olfactory perception is a complicated phenomenon in which molecular interactions play a crucial role. Dimopoulou et al. [34] suggested for the first time that the bacterial EPSs produced in wine could contribute to aromatic complexity and fruity aromas. These may affect the release of esters and alcohols from the liquid phase, affecting their concentration in the head space [113]. Indeed, the wine matrix plays an essential role in the in vivo aroma release [114]. When dextrans from *Lc. mesenteroides* were added to wine model medium, isoamyl acetate and ethyl hexanoate esters were salted out in the vapor phase as the presence of polysaccharides decreased their solubility [115]. Bastard et al. [36] also suggest an interaction between the biofilm formed on the oak surface and wine, contributing to the expression of trans-whisky lactone complexity.

## 4. Conclusions

The ability of LAB to produce EPSs in wine and other fermented beverages has been known for a long time, but mainly in the context of ropy β-glucan accumulation and wine spoilage. Great genotypic and phenotypic diversity within LAB EPS producers, especially *O. oeni*, has been more recently elucidated. The more the research advances, the more the complexity of EPS biosynthesis in fermented beverages is unraveled, revealing the multiple biosynthetic pathways and the associated phenotypic diversity. The effects of the EPSs produced on bacterial survival and dissemination, as well as on wine quality, are complex and trigger many fascinating research perspectives for the future. An important question is whether the EPSs produced by LAB of fermented beverages ultimately have a positive or negative effect. No one-word answer to this question yet exists, and this will certainly require many years of work.

## Figures and Tables

**Table 1:** LAB EPSs isolated from fermented beverages.

EPS Type	EPS Structure	Species	Niche	Implicated Genes	Consequences/Role of EPS	Reference
homopolysaccharides	β-glucan	*Oenococcus oeni*	wine, cider	*gtf*	ropy character, stress resistance	[17,18,19]
	β-glucan	*Pediococcus damnosus*	cider	*gtf*	ropy character	[17,20,21,22,23]
	β-glucan	*Pediococcus parvulus*	cider, wine	*gtf*	ropy character, stress resistance	[18,23]
	β-glucan	*Pediococcus ethanolidurans*	cider	-	ropy character	[24]
	β-glucan	*Pediococcus claussenii*	beer	*gtf*	ropy character	[25,26]
	β-glucan	*Lactobacillus brevis*	beer	*gtf2*	ropy character, ethanol tolerance, biofilm formation	[26,27]
	β-glucan	*Lactobacillus diolivorans*	cider	*gtf*	-	[17]
	β-glucan	*Lactobacillus suebicus*	cider	*gtf*	ropy character	[28,29]
	β-glucan	*Lactobacillus* spp.	cider	-	ropy character	[30]
	a-glucan	*Leuconostoc pseudomesenteroides*, *Weissella confusa*	beer	*dsr*	increased viscosity	[31]
	dextran	*Leuconostoc pseudomesenteroides*	homemade wine	-	-	[32]
	glucan and fructan	*Leuconostoc mesenteroides*	grape must and wine	Glucosyltransferase gene	more or less mucoid strains	[33]
	dextran and levan	*Oenococcus oeni*	wine	*dsrO* and *levO*	lyoprotective ability to freeze-drying process	[15,34,35]
heteropolysaccharides	glucose, galactose, rhamnose	*Oenococcus oeni*	wine	*eps* cluster	aromatic complexity, biofilm formation, capsule, lyoprotective ability to freeze-drying	[15,34,35,36]
	glucose, galactose, galactofuranose	*Lactobacillus suebicus*	cider	*gtf*	ropy character	[28,29]
	glucose, galactose, *N*-*acetyl*-glucosamine, phosphate	*Lactobacillus suebicus*	cider	*eps* cluster	ropy character	[37]
	glucose, galactose, glucosamine	*Pediococcus ethanolidurans*	cider	-	ropy character	[24]
